# Novel human polyomaviruses, Merkel cell polyomavirus and human polyomavirus 9, in Japanese chronic lymphocytic leukemia cases

**DOI:** 10.1186/1756-8722-5-25

**Published:** 2012-06-01

**Authors:** Masayuki Imajoh, Yumiko Hashida, Ayuko Taniguchi, Mikio Kamioka, Masanori Daibata

**Affiliations:** 1 Department of Microbiology and Infection, Kochi Medical School, Kochi University, Nankoku, Kochi, 783-8505, Japan; 2 Department of Hematology and Respiratory Medicine, Kochi Medical School, Kochi University, Nankoku, Kochi, 783-8505, Japan; 3 Department of Laboratory Medicine, Kochi Medical School, Kochi University, Nankoku, Kochi, 783-8505, Japan

**Keywords:** Chronic lymphocytic leukemia, Merkel cell polyomavirus, Human polyomavirus 9, Japanese study

## Abstract

**Background:**

Chronic lymphocytic leukemia (CLL) is the rarest adult leukemia in Japan, whereas it is the most common leukemia in the Western world. Recent studies from the United States and Germany suggest a possible etiological association between Merkel cell polyomavirus (MCPyV) and CLL, although no data have been reported from Eastern countries. To increase the volume of relevant data, this study investigated the prevalence and DNA loads of MCPyV and human polyomavirus 9 (HPyV9), another lymphotropic polyomavirus, in Japanese CLL cases.

**Findings:**

We found that 9/27 CLL cases (33.3 %) were positive for MCPyV using quantitative real-time polymerase chain reaction analysis. The viral DNA loads ranged from 0.000017 to 0.0012 copies per cell. All cases were negative for HPyV9. One MCPyV-positive CLL case was evaluated by mutational analysis of the *large T* (*LT*) gene, which indicated the presence of wild-type MCPyV without a nucleotide deletion. DNA sequence analysis of the entire *small T* (*ST*) gene and the partial *LT* gene revealed that a Japanese MCPyV isolate, designated CLL-JK, had two nucleotide gaps when compared with the reference sequence of the North American isolate MCC350.

**Conclusions:**

This study provides the first evidence that MCPyV is present in a subset of Japanese CLL cases with low viral DNA loads. MCPyV and HPyV9 are unlikely to contribute directly to the development of CLL in the majority of Japanese cases. MCPyV isolated from the Japanese CLL cases may constitute an Asian group and its pathogenicity needs to be clarified in future studies.

## Introduction

Chronic lymphocytic leukemia (CLL) is the most common form of adult leukemia in the Western countries, where its frequency is ca. 3–8 new cases per 100,000 persons per year [1-3]. However, CLL is very rare in far Eastern countries. The incidence of CLL in Japan is 0.48 per 100,000 persons per year, although the proportion of T-CLL is relatively high [4]. It has been suggested that genetic predisposition is a causal factor in CLL, but other largely unknown etiological factors may contribute to the geographical difference in the CLL frequency. Infectious agents may be correlated directly or indirectly with the pathogenicity of CLL, including viral infections.

Polyomaviruses are nonenveloped icosahedral viruses with a circular double-stranded DNA genome that encodes certain oncoproteins and they are putative oncogenic viruses [5]. Currently, nine human polyomaviruses are known but their precise roles in carcinogenesis remain poorly understood [6]. The fifth polyomavirus, Merkel cell polyomavirus (MCPyV), was reported in 2008 by Feng et al. [7]. It was detected in approximately 80 % of Merkel cell carcinomas (MCCs), which is an aggressive neuroendocrine skin carcinoma. Human polyomavirus 9 (HPyV9) was identified recently in the serum of kidney transplant patients who were receiving immunosuppressive treatment [8]. The presence of the HPyV9 genome in particular diseases has not been clarified. MCPyV and HPyV9, with a close phylogenetic relationship to the African green monkey-derived lymphotropic polyomavirus, have the potential to infect lymphoid cells [8,9].

The search for hematologic neoplasias where MCPyV has a role in etiopathogenesis is currently an important issue [10]. The possible association of MCPyV with various lymphoid malignancies was first demonstrated by US researchers, but they did not find a significant association between CLL and MCPyV [11,12]. However, Canadian and German studies detected MCPyV-positive CLL cases and suggested the possible involvement of MCPyV in a subset of CLLs [13-15]. More recently, another US group reported MCPyV-positive CLLs with very low MCPyV copy numbers [16]. Thus, all investigations of links between MCPyV and CLL have come from the Western world, whereas the prevalence of MCPyV in CLL cases in Eastern countries has not been investigated.

In this study, we aimed to determine the prevalence and DNA loads of MCPyV and HPyV9 in Japanese CLL cases, and we provide the first findings of a relationship between CLL and new human polyomaviruses in the Eastern world.

## Methods

### Patients and DNA preparation

We recruited 27 Japanese patients with CLL, including 25 B-CLLs and 2 T-CLLs (cases 3 and 17). The cohort comprised 18 males and nine females with a median age of 63 years at diagnosis (range 49–93). We also studied peripheral blood samples from 18 healthy Japanese donors with a median age of 48 years (range 27–55). A serum fraction containing abundant leukemia cells was separated using Ficoll–Conray density gradient and frozen at −80°C until use. Total DNA was extracted using the standard phenol–chloroform method. Approval was obtained from the ethics committee for this study.

### Quantitative real-time polymerase chain reaction (PCR)

Quantitative real-time PCR was conducted with 500 ng of extracted DNA, according to the method of Bhatia et al. [17] with some modifications. The primer and probe sequences are shown in Table 1. The primers and probe used for detecting the *VP1* gene of HPyV9 were designed based on the GenBank sequence NC015150. Standard PCR was conducted using the same primers and the PCR product was cloned into the pMD20-T vector (TaKaRa, Shiga, Japan). We prepared six-fold serial dilutions using 10 ng of the cloned plasmid DNA to generate a standard curve and we calculated the copy number in each sample. The viral DNA load was defined as the viral DNA copies per *RNase P* gene copy, which represented the copy number per cell.

**Table 1:** Primer sequences used in this study

**Real-time PCR analysis**			
**Target gene**	**Forward (5′ → 3′)**	**Reverse (5′ → 3′)**	**Probe (5′ → 3′)**
MCPyV *ST*	GCAAAAAAACTGTCTGACGTGG	CCACCAGTCAAAACTTTCCCA	FAM-TATCAGTGCTTTATTCTTTGGTTTGGATTTCCTCCT- TAMRA
HPyV9 *VP1*	TGCTGTTGATATTGTTGGAATTCA	AACAACCCGTTTCCTTAGAGTTACA	FAM-CTGGAGAGGCCTACCT-NFQ-MGB
Human *RNase P*	AGATTTGGACCTGCGAGCG	GAGCGGCTGTCTCCACAAGT	FAM-TTCTGACCTGAAGGCTCTGCGCG-TAMRA

**PCR analysis**			
**Target gene (positions)**	**Forward (5′ → 3′)**	**Reverse (5′ → 3′)**	
MCPyV *LT* (1867–2221)	AGCCCCCTTACAAATTACTGCAAG^*^	AGCATTTCTGTCCTGGTCATTTC^*^	
MCPyV *ST-LT* (183–828)	GCATATAGACAAGATGGATTTA^†^	ATAACCTTTCTTTGATATTTTGC^†^	
MCPyV *ST-LT* (571–1157)	TTGTCTCGCCAGCATTGTAG^†^	GGATCCAGAGGATGAGGTGGGTTC^†^	

^*^Primers for mutational analysis.

^†^Primers for sequence analysis of the *ST-LT* genes.

### PCR and DNA sequence analysis

MCPyV-positive samples identified by real-time PCR amplification were subjected to mutational analysis of the *large T* (*LT*) gene using standard PCR, as reported by Pantulu et al. [14]. The primers shown in Table 1 yielded a 355-bp PCR product representing wild-type MCPyV, while the 120-bp product represented mutated MCPyV with a nucleotide deletion [14].

Nucleotide sequences at positions 183 to 1157 (based on the GenBank sequence EU375803), including the entire *small T* (*ST*) gene and the partial *LT* gene, were also investigated by standard PCR for 35 cycles using two overlapping primer sets (nucleotide 183 to 828 and nucleotide 571 to 1157) (Table 1). We expected amplicons of 646 bp and 587 bp with these PCR amplifications. The PCR products were gel purified and directly sequenced with a Big Dye Terminator Cycle Sequencing Kit (Life Technologies, Tokyo, Japan) on a 3130 Genetic Analyzer Instrument (Applied Biosystems, Tokyo, Japan). The nucleotide sequences that we obtained were analyzed using the BioEdit program and deposited in GenBank under accession number AB709861. The DNA sequence data were compared with reference sequences of MCPyV isolates from the National Center for Biotechnology Information Entrez Nucleotide database (MCC350, MCC339, MKL-1, TKS, and 16b) and from a previous report (B.C.) [18].

## Results and discussion

We found that 9/27 CLL cases (33.3 %) were positive for MCPyV according to the real-time PCR analysis (Table 2). Previous studies conducted in Canada, Germany, and the USA detected MCPyV in 20.8 % (5/24), 27.1 % (19/70), and 33.3 % (6/18) of CLL cases, respectively [13,14,16]. Thus, our MCPyV detection rate was similar to that reported from Western countries. The DNA loads of the nine MCPyV-positive cases ranged from 0.000017 to 0.0012 copies per cell (Table 2), which was consistent with previous data from North American CLL cases where viral DNA loads were < 0.0004 [16]. Our real-time PCR analysis showed that the copy numbers of nine MCPyV-positive Japanese MCC controls ranged from 1.25 to 8.32 (data not shown). Thus, the MCPyV-positive Japanese CLL cases had 3–5 log lower copy numbers compared with the MCPyV-positive Japanese MCCs. One of the 18 peripheral blood samples from healthy Japanese donors was positive for MCPyV with 0.00015 copies per cell (data not shown). A two-tailed Fisher's exact test with a significant level of *P* < 0.05 showed that the MCPyV detection rate in CLL patients was significantly higher than that in healthy individuals (*P* = 0.034). However, Shuda et al. [11] proposed that samples with < 0.001 copies per cell should be considered MCPyV-negative when determining the frequency of MCPyV in lymphomas. Thus, all of our cases would be negative for MCPyV, except case 19, if we applied their detection limit. Recently, Pancaldi et al. [18] reported that MCPyV was also detectable in the buffy coats of healthy Italian blood donors whose viral DNA loads ranged from 0.00001 to 0.0001 copies per cell. Overall, we found that our CLL cases harbored only low-level viral loads, so this study provided little support for a direct causal role of MCPyV in the majority of Japanese CLL cases.

**Table 2:** DNA loads of MCPyV and HPyV9 detected by quantitative real-time PCR

**Case**	**Viral DNA load (copies per cell)**		**Case**	**Viral DNA load (copies per cell)**	
	**MCPyV**	**HPyV9**		**MCPyV**	**HPyV9**
**1**	-	-	**15**	-	-
**2**	-	-	**16**	-	-
**3**	-	-	**17**	-	-
**4**	0.000034	-	**18**	0.00041	-
**5**	-	-	**19**	0.0012	-
**6**	-	-	**20**	0.00041	-
**7**	-	-	**21**	0.000039	-
**8**	-	-	**22**	0.000051	-
**9**	-	-	**23**	-	-
**10**	-	-	**24**	0.000017	-
**11**	-	-	**25**	0.00061	-
**12**	-	-	**26**	0.000027	-
**13**	-	-	**27**	-	-
**14**	-	-			

We tested the nine MCPyV-positive cases to determine the presence of truncating mutations of the *LT* gene, which have been reported previously [14]. However, *LT* sequences were not detectable in all samples by standard PCR, possibly because of the low viral DNA loads. The 355-bp amplicon was only detected in case 21 without the nucleotide deletion, indicating a wild-type MCPyV infection in this case. Pantulu et al. [14] detected the mutated *LT* gene in 6/19 MCPyV-positive German CLL patients, but it is uncertain whether such truncating *LT* mutations are prevalent in Japanese CLL cases.

**Figure 1:**
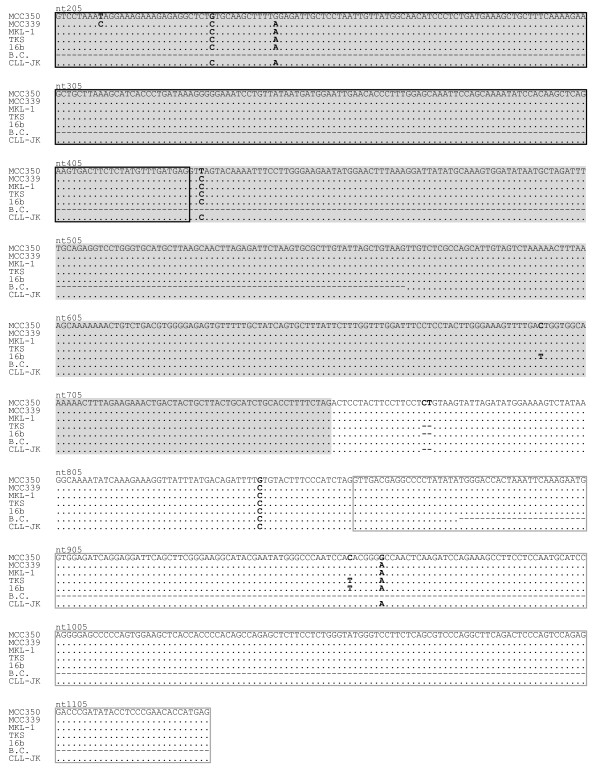
**Nucleotide comparison of the MCPyV isolate, designated CLL-JK, with the reference isolates.** Regions coding the *ST* gene are represented as gray boxes. The gray boxes with black lines and white boxes with gray lines correspond to exon 1 and exon 2 of the *LT* gene, respectively. Nucleotide substitutions are shown in bold and compared with the nucleotide sequence of MCC350.

We also investigated the DNA sequences of the MCPyV *STLT* genes at positions 183 to 1157. The 646-bp and 587-bp products were amplified in five cases, i.e., cases 18, 19, 21, 22, and 25. The sequencing results for the PCR amplicons showed that their nucleotide sequences were identical. The sequences found in our CLL cases, designated CLL-JK, shared two nucleotide gaps at positions 774 and 775 with sequences from another Japanese isolate, TKS [GenBank: FJ464337] [19], and Asian isolate 16b [HM011548] [9], when compared with the sequence of North American isolate MCC350 [EU375803] (Figure 1). Touzé et al. [20] reported that the nucleotide sequences of French MCPyV isolates were different from the MCC350 isolate, but homologous to the Swedish isolate MKL-1 [FJ173815]. A recent study also showed that the nucleotide sequence of the Italian isolate B.C. shared 100 % homology with MKL-1 [18]. It is likely that the MCPyV strains circulating in Europe are genetically conserved. Thus, the CLL-JK isolate with nucleotide gaps, TKS, and 16b, may be classified with the Asian group, which is distinct from the European and North American groups. It is notable that a C to T substitution was common in the TKS and 16b isolates at position 958 in the exon 2 region of the *LT* gene (Figure 1), which resulted in an amino acid substitution of His to Tyr. It is not known whether the genetic diversity of the Asian group affects the pathogenicity of MCPyV.

We also aimed to determine whether the lymphotropic polyomavirus HPyV9 was associated with CLL. We found that no cases with CLL or healthy donors had amplifiable HPyV9 DNA according to the highly sensitive real-time PCR (Table 2). Our results appear to indicate that HPyV9 is not involved directly in the leukemogenesis of CLL.

In summary, this study provides the first evidence of MCPyV DNA in a subset of Japanese CLL cases. Mutational analysis of the MCPyV *LT* gene was not fully evaluated because of low viral DNA levels, but one MCPyV-positive CLL case had wild-type MCPyV. These findings suggest that MCPyV is unlikely to have a direct causal role in the majority of Japanese CLL cases. DNA sequence analysis revealed that the MCPyV isolates found in our CLL patients constituted an Asian group with two nucleotide gaps. We also showed that HPyV9 was not present in Japanese CLL cases. Further worldwide epidemiological and virological studies are required to determine the pathogenetic relevance of human polyomaviruses in CLL.

## Competing interests

The authors declare that they have no competing interests.

## Authors’ contributions

MI carried out the PCR analysis and DNA sequencing, analyzed the data and drafted the manuscript. YH carried out the PCR analysis and DNA sequencing. AT and MK collected the samples. MD conceived the study, collected the samples, contributed to the acquisition of funding and revised the manuscript. All authors read and approved the final manuscript.
